# Gut Microbial Composition and Short-Chain Fatty Acid Metabolism in Cognitively Unimpaired Adults Stratified by Amyloid-β Status

**DOI:** 10.3390/biom16010018

**Published:** 2025-12-22

**Authors:** D. M. Sithara Dissanayaka, Thilini N. Jayasinghe, Hamid R. Sohrabi, S. R. Rainey-Smith, Kevin Taddei, Colin L. Masters, Ralph N. Martins, W. M. A. D. Binosha Fernando

**Affiliations:** 1 Centre of Excellence for Alzheimer’s Disease Research & Care, School of Medical and Health Sciences, Edith Cowan University, Joondalup, WA 6027, Australia; sitharad@our.ecu.edu.au (D.M.S.D.);; 2 Alzheimer’s Research Australia, Ralph and Patricia Sarich Neuroscience Research Institute, Nedlands, WA 6009, Australia; 3 The Charles Perkins Centre, The University of Sydney, Camperdown, NSW 2006, Australia; 4 Centre for Healthy Ageing, Murdoch University, Murdoch, WA 6150, Australia; 5 Department of Biomedical Sciences, Faculty of Medicine, Health and Human Sciences, Macquarie University, Sydney, NSW 2109, Australia; 6 The Florey Institute of Neuroscience and Mental Health, University of Melbourne, Melbourne, VIC 3052, Australia

**Keywords:** Short-chain fatty acids (SCFAs), Gut microbiome, Amyloid-β (Aβ) pathology, Shotgun metagenomics, Preclinical Alzheimer’s disease

## Abstract

Short-chain fatty acids (SCFAs) produced by gut microbial fermentation influence host metabolism and neuroinflammatory processes implicated in Alzheimer’s disease (AD). However, the relationship between fecal SCFAs, microbial taxa, and cerebral amyloid-β (Aβ) burden in cognitively unimpaired individuals remains unclear. Fecal SCFAs were quantified using GC-MS, and microbial species were profiled by shotgun metagenomics in 87 participants. Associations between SCFAs, demographics, APOE ε4 status, and Aβ burden were tested using nonparametric statistics and multivariable regression. Microbial–SCFA links were evaluated using Spearman correlations and multivariate ordinations, with mediation analysis exploring potential indirect pathways. Acetate was the predominant SCFA and demonstrated the most robust microbial associations. Higher acetate concentrations were positively associated with *Bacteroides ovatus* and *Faecalibacterium prausnitzii*, whereas lower acetate levels were linked to species such as *Bifidobacterium animalis* and *Lachnoclostridium scindens*. Stratified analyses indicated that individuals with elevated Aβ burden exhibited more pronounced species–SCFA relationships, including a notable association between *Bacteroides thetaiotaomicron* and butyrate. Multivariate ordination further identified a significant overall coupling between SCFA profiles and microbial community structure. Mediation analysis suggested that an *Oscillospiraceae* species may represent a potential intermediary linking valerate concentrations with Aβ status. SCFA concentrations were not strongly influenced by demographic or genetic factors, but specific species demonstrated robust associations with acetate levels. Distinct SCFA–microbial interaction patterns in Aβ High individuals suggest subtle early gut microbial alterations linked to amyloid burden. These findings highlight the potential role of SCFA-related microbial pathways in preclinical AD.

## 1. Introduction

The gut microbiome influences host physiology through its metabolites, particularly short-chain fatty acids (SCFAs), which are produced via microbial fermentation of dietary substrates. SCFAs, including acetate, propionate, and butyrate, act as key mediators of host–microbe communication by regulating energy metabolism, inflammation, and neuroimmune function through the gut–brain axis [1,2,3]. Studies have demonstrated associations in Alzheimer’s disease (AD); however, investigations focusing on these relationships during the preclinical stage, when amyloid accumulation occurs in cognitively unimpaired individuals, remain limited.

Experimental studies suggest that SCFAs exert multiple neuroprotective effects. Butyrate enhances neurotrophic factor expression and protects against amyloid-induced neurotoxicity, while propionate and acetate influence microglial activation, systemic inflammation, and blood–brain barrier integrity [4,5]. Disruptions in SCFA production arising from dietary imbalance, microbial dysbiosis, or aging may therefore contribute to neurodegenerative processes associated with AD [6].

Metagenomic studies have revealed reductions in SCFA-producing taxa, such as *Faecalibacterium prausnitzii* and *Roseburia* spp., alongside increases in pro-inflammatory species like *Lachnoclostridium* and *Enterobacteriaceae* [7,8]. This may promote a metabolically altered and inflammatory gut environment. These compositional imbalances have been reported in individuals with AD and mild cognitive impairment, but their occurrence in the preclinical stage of disease remains less well understood [6]. Importantly, the specific relationships between individual microbial species and fecal SCFA concentrations and how these vary with cerebral amyloid burden have yet to be identified. While several studies have examined microbiome composition or SCFA concentrations independently, few have integrated both datasets to assess how specific microbial species contribute to SCFA production across varying amyloid stages [9,10]. Furthermore, given that both microbial metabolism and amyloid pathology are influenced by age, sex, and APOE ε4 genotype, understanding these interconnections in cognitively unimpaired adults is important for identifying early gut-related biomarkers of AD risk [11,12,13].

This study addresses these gaps by investigating fecal SCFA concentrations and gut microbial composition in a subset of AIBL WAMS participants who are cognitively unimpaired but stratified by cerebral Aβ status. Using shotgun metagenomic sequencing and gas chromatography–mass spectrometry (GC-MS), the study explores correlations between microbial species and SCFA concentrations based on amyloid level. Then, this study integrates findings through multivariate and network-based analyses. The overarching aim is to identify microbial taxa contributing to SCFA variability and to determine whether these associations differ in individuals with higher amyloid burden at the preclinical stage. These insights may help clarify the role of the gut microbiome in early AD and support the development of microbiome-based strategies for prevention and early intervention.

## 2. Methodology

### 2.1. Participant Selection

Participants were recruited from two extensively characterized Australian aging cohorts: the Australian Imaging, Biomarkers, and Lifestyle (AIBL) Study and the Western Australian Memory Study (WAMS). These cohorts were selected because they provide rich clinical, cognitive, neuroimaging, and biomarker data, enabling detailed investigation of gut microbial alterations associated with early AD. The AIBL study is a large, longitudinal cohort designed to identify biological and lifestyle determinants of AD [14], while WAMS focuses on cognitive aging, memory trajectories, and risk factors for neurodegeneration [15].

This study examined a cross-sectional design to explore microbial features associated with preclinical and symptomatic stages of AD. A total of 87 cognitively unimpaired older adults who provided written informed consent for fecal sample collection were included. Group stratification was based on cerebral amyloid-beta (Aβ) status, determined using PET imaging conducted prior to stool sampling. Participants were classified as either Aβ Low (*n* = 68), representing individuals with low cerebral Aβ burden, or Aβ High (*n* = 19), representing those with elevated Aβ deposition. For analytical purposes, individuals with one or two APOE ε4 alleles (ε3/ε4 or ε4/ε4) were combined and classified as APOE4 carriers, and those with no ε4 alleles were classified as non-carriers. The cognitively unimpaired (CU) Aβ High subgroup was relatively small, reflecting the rarity of this population, as only about 20–25% of cognitively unimpaired older adults are amyloid-positive. Obtaining such well characterized preclinical cases is inherently challenging, and their inclusion in this study represents a unique strength, offering rare early insight into microbiome configurations associated with amyloid pathology and providing a foundation for future larger-scale investigations.

### 2.2. Ethics

Fecal sample collection for the AIBL cohort (2006/ETH/0215; approval date: 6 December 2022) and WAMS cohort (2003/ETH/0139; approval date: 23 July 2023) adhered to ethical requirements approved by the Ramsay Health Care WA/SA Human Research Ethics Committee (Australia). Data management procedures were approved by the Edith Cowan University Human Research Ethics Committee (REMS NO: 2023-04565-DISSANYAKA).

### 2.3. Classification of CU Aβ High and CU Aβ Low Participants

Amyloid burden was quantified using PET imaging with one of five tracers: ^11^C-Pittsburgh compound B, ^18^F-flutemetamol, ^18^F-florbetapir, ^18^F-florbetaben, or ^18^F-NAV4694, following previously standardized imaging protocols [16]. Centiloid (CL) values were derived using the CapAIBL pipeline [16], offering a continuous measure of global Aβ deposition. CU participants with CL values ≤ 15 were classified as Aβ Low, whereas those with CL > 15 were categorized as Aβ High [14,17].

### 2.4. Determination of MCI and AD Diagnoses

MCI and AD were diagnosed according to established clinical criteria. AD classification followed accepted diagnostic frameworks, while MCI was defined by objective cognitive impairment in the absence of significant functional decline, consistent with earlier descriptions [14].

### 2.5. Fecal Sample Quantification

#### 2.5.1. Sample Processing

Stool samples were collected using DNA/RNA Shield Fecal Collection Tubes (Zymo Research, Irvine, CA, USA) under a standardized protocol. Participants collected a morning stool sample, stored it on ice during transport, and delivered it to the Centre of Excellence for Alzheimer’s Research Australia (Nedlands, Perth) within two hours. Samples were subsequently stored at −80 °C until analysis.

#### 2.5.2. Short-Chain Fatty Acid (SCFA) Extraction and GC-MS Quantification

Fecal short-chain fatty acids (acetic, propionic, butyric, isobutyric, isovaleric, and valeric acids) were quantified using gas chromatography–mass spectrometry (GC-MS). Approximately 1 g of frozen stool was extracted in acidified methanol containing an internal standard (2-ethylbutyric acid). Samples were homogenized and centrifuged, and the clarified supernatant was filtered (0.22 µm) before analysis. SCFAs were measured on a Thermo Scientific ISQ LT GC-MS system using a BP20 capillary column under standardized temperature-gradient conditions. Compounds were identified by retention time and mass spectra, and quantification was performed using multi-point calibration curves (R^2^ > 0.998). Final concentrations were calculated in mmol/kg of fecal material.

### 2.6. DNA Extraction

Shotgun metagenomic sequencing was performed on all fecal DNA samples to enable high-resolution species-level characterization of the gut microbiome. Genomic DNA was extracted from fecal samples using the DNeasy PowerSoil Pro Kit (QIAGEN, Germantown, MD, USA) following the manufacturer’s protocol. DNA quantity and purity were assessed using fluorometric and spectrophotometric methods. Metagenomic libraries were prepared with the Illumina DNA Prep (M) Tagmentation Kit and sequenced on the Illumina NovaSeq X Plus platform (2 × 150 bp). The mean sequencing depth was 21.5 million paired-end reads per sample.

### 2.7. Metagenomic Library Preparation and Sequencing

Metagenomic libraries were prepared using the Illumina DNA Prep (M) Tagmentation Kit with unique dual indexing and sequenced at the Australian Genome Research Facility (AGRF). Libraries were sequenced on the Illumina NovaSeq X Plus platform (Illumina, San Diego, CA, USA) using 2 × 150 bp paired-end chemistry. High-depth shotgun metagenomic sequencing was performed to enable species-level taxonomic profiling and downstream microbial analyses. The mean sequencing depth was approximately 21.5 million reads per sample. Primary base calling and FASTQ file generation were performed using standard DRAGEN BCL Convert software v 4.0.3 (Illumina, San Diego, CA, USA), and sequencing quality metrics were assessed using MultiQC tool (v1.11) [18].

### 2.8. Bioinformatics Processing

Quality filtering and adapter trimming were performed with Trim Galore v0.4.5 (Babraham Bioinformatics, Cambridge, UK), removing low-quality bases (Phred < 20) [19]. Post-QC, 99.95% of reads were retained, with a minimum depth of 3.92 million reads per sample, ensuring sufficient coverage for downstream taxonomic profiling.

### 2.9. Data Filtering and Integration

#### 2.9.1. Taxonomic Profiling

High-quality reads were taxonomically classified using Kraken2 (v2.0.8), and species-level abundance was estimated with Bracken [20,21]. Classification was performed across all major taxonomic ranks to obtain a comprehensive overview of the microbial community. Non-bacterial taxa (Archaea, Eukaryotes, and viruses) were removed prior to analysis. A prevalence threshold of 10% was applied to retain taxa present in at least 10% of samples, thereby minimizing noise from low-abundance features. Additional refinement steps included removing duplicate, unclassified, uncultured, and low-variance taxa. After normalization and filtering, the final taxonomic dataset comprised 20 phyla, 53 classes, 136 orders, 316 families, 1116 genera, and 2998 species.

#### 2.9.2. Species-Level Refinement

Species present in at least 10% of participants and with a total read count of ≥100 were retained, resulting in 103 species for downstream analyses. This refinement step minimized noise from low-prevalence or low-abundance taxa, ensuring that subsequent SCFA-related analyses focused on consistently represented and biologically relevant species.

#### 2.9.3. Data Harmonization

SCFA and microbiome datasets were matched by participant ID. Only individuals with complete paired data were retained (*n* = 87).

### 2.10. Statistical Analyses

#### 2.10.1. Descriptive Analyses (Whole Dataset; Compared by Amyloid Stage)

Descriptive statistics were used to summarize participant demographics and fecal SCFA concentrations across the full cohort (*n* = 87). Group comparisons for Aβ status, sex, and APOE4 genotype were performed using Wilcoxon rank-sum tests, and associations with age were examined using Spearman correlation. False discovery rate (FDR) correction was applied to adjust for multiple testing, with q < 0.05 considered significant. All analyses were conducted in R v4.4.1 (R Foundation for Statistical Computing, Vienna, Austria).

#### 2.10.2. Multivariable Regression Analyses of SCFA Concentrations (Whole Dataset)

Multivariable linear regression models were used to examine the independent associations between fecal SCFA concentrations and participant variables. Each model included one SCFA (acetic, propionic, isobutyric, butyric, isovaleric, valeric, or total SCFAs) as the dependent variable, with age, sex, APOE4 carrier status, and Aβ status entered as simultaneous predictors. Model residuals were checked for normality, and results were adjusted for multiple comparisons using the Benjamini–Hochberg false discovery rate (FDR) method, with q < 0.05 considered significant.

#### 2.10.3. Stratified and Interaction Analyses (Amyloid-Stage and Demographic Subgroups)

To determine whether relationships between microbes and SCFAs differ by biological context, analyses were stratified by:

##### Aβ Status (Aβ Low vs. Aβ High)

Differences in fecal SCFA concentrations between Aβ Low and Aβ High participants were assessed using the Wilcoxon rank-sum test. To account for multiple comparisons across SCFAs, false discovery rate (FDR) adjustment was applied using the Benjamini–Hochberg method, with q-values < 0.05 considered statistically significant.

##### Sex (Male vs. Female)

SCFA concentrations were compared between male and female participants using Wilcoxon rank-sum tests. Multiple comparisons were corrected using the Benjamini–Hochberg FDR (q < 0.05) method.

##### APOE ε4 Carrier Status (Carrier vs. Non-Carrier)

SCFA concentrations were compared between APOE ε4 carriers and non-carriers using Wilcoxon rank-sum tests. Multiple comparisons were adjusted using the Benjamini–Hochberg false discovery rate (FDR) method, with q-values < 0.05 considered statistically significant.

##### Age Groups

Associations between participant age (as a continuous variable) and fecal SCFA concentrations were examined using Spearman’s rank correlation. Multiple testing was addressed using the Benjamini–Hochberg false discovery rate (FDR) method, with q-values < 0.05 indicating statistical significance.

#### 2.10.4. Correlation Between Gut Microbial Species and SCFA Concentrations (Whole Dataset and Stratified by Amyloid Stage)

To explore associations between bacterial species and fecal SCFA levels, Spearman’s rank correlation analyses were conducted for each SCFA. Analyses were performed across the full dataset and separately within Aβ Low and Aβ High groups to identify both overall and amyloid-specific microbial–metabolite relationships. *p*-values were adjusted for multiple testing using the Benjamini–Hochberg false discovery rate (FDR) method, with statistical significance defined as q < 0.05. Associations not surviving FDR correction but showing unadjusted *p* < 0.05 were considered indicative of potential trends.

#### 2.10.5. Species–SCFA Correlations Stratified by Aβ Status

Pairwise Spearman’s rank correlation analyses were performed to assess associations between the relative abundances of 103 microbial species and fecal SCFA concentrations. Analyses were conducted separately within Aβ High and Aβ Low groups to identify group-specific microbial–metabolite relationships. Correlation coefficients (ρ) and *p*-values were computed using the Hmisc package in R, and significance was evaluated at *p* < 0.05. False discovery rate (FDR) correction was applied within each group using the Benjamini–Hochberg method (q < 0.05 considered significant).

#### 2.10.6. Multivariate Pattern Recognition and Clustering (Whole Dataset; Amyloid as Grouping Factor)

To explore overall patterns in the combined microbial-species and SCFA dataset, principal component analysis (PCA) and principal coordinates analysis (PCoA) were performed on centered log-ratio (CLR) transformed data. Euclidean distance was used for PCoA construction. Plots were colored by Aβ status (Aβ High vs. Aβ Low), and 68% confidence ellipses were added to visualize within-group variability. Permutational multivariate analysis of variance (PERMANOVA, 999 permutations) assessed whether overall compositional profiles differed significantly between Aβ groups while accounting for age, sex, and APOE genotype. All analyses were conducted in R (version 4.3.3) using the vegan, compositions, and *ggplot2* packages.

#### 2.10.7. Network-Based Microbial–Metabolite Integration (Amyloid-Stratified)

Network visualizations were considered to summarize significant correlations (FDR < 0.05) between fecal SCFAs and microbial species across Aβ High and Aβ Low groups. Nodes represent SCFAs or bacterial species, and edges represent positive or negative associations. These exploratory analyses informed subsequent multi-omics network integration.

#### 2.10.8. Canonical Correlation Analysis (CCA)

CCA was applied to identify multivariate relationships between microbial community composition and SCFA profiles. A partial canonical correspondence analysis (pCCA) was performed using the vegan package in R, constraining the ordination by SCFA concentrations while adjusting for age, sex, APOE ε4 status, and Aβ status through the Condition() function. Model significance was assessed using 999 permutations, and 95% confidence ellipses were plotted to visualize group separation between Aβ High and Aβ Low participants.

#### 2.10.9. Mediation Analyses (Amyloid-Stratified Causal Framework)

To evaluate whether microbial taxa mediate the relationship between fecal SCFA concentrations and Aβ status (High vs. Low), causal mediation analysis was performed using the mediation R package. Each model included SCFAs as the exposure, taxon as the mediator (filtered by ≥10% prevalence and ≥100 reads), and Aβ status as the outcome. Covariates were age, sex, and APOE ε4 status. The average causal mediation effect (ACME), direct effect (ADE), total effect, and proportion mediated were estimated using nonparametric bootstrapping (1000 simulations). Statistical significance was defined as *p* < 0.05 for ACME, ADE, and total effects.

### 2.11. Multiple Testing and Visualization

All statistical analyses were adjusted for multiple comparisons using the Benjamini–Hochberg false discovery rate (FDR) method, with q < 0.05 considered statistically significant. Data visualizations were generated in R using standard visualization packages.

## 3. Results

### 3.1. Descriptive Analyses (Whole Dataset; Compared by Amyloid Stage)

Descriptive statistics summarized participant demographics and SCFA concentrations across the cohort (*n* = 87) (Table 1). CU Aβ High participants were significantly older than those in the CU Aβ Low group (81 vs. 76 years, *p* = 0.019), and a higher proportion were APOE4 carriers (58.3% vs. 13.2%, *p* = 0.026). Sex distribution, education, and BMI did not differ significantly between groups. All seven SCFAs showed comparable concentrations between Aβ groups, with no significant group differences (all *p* > 0.40).

### 3.2. Multivariable Regression Analyses of SCFA Concentrations

**Figure 1:**
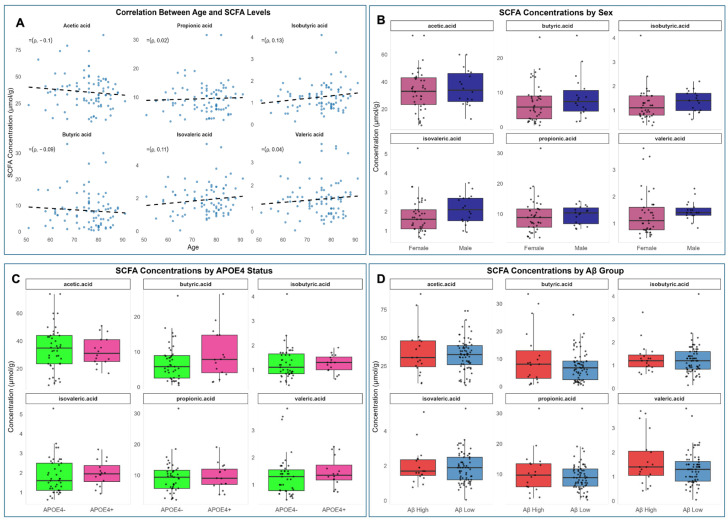
Association of short-chain fatty acid (SCFA) concentrations by age, sex, APOE4 status, and cerebral amyloid-β (Aβ) status. (**A**) Scatterplots showing correlations between age and concentrations of six SCFAs. (**B**–**D**) Boxplots showing SCFA concentrations stratified by sex (**B**), APOE4 status (**C**), and Aβ group (**D**). Each plot displays the median, interquartile range, 1.5× IQR whiskers, and individual data points. Nominal *p*-values (*p* < 0.05) were observed for butyric and valeric acids (sex), and for acetic acid (Aβ group), although none of these associations remained statistically significant after FDR correction (q > 0.20).

Multivariable linear regression models examined independent associations of fecal SCFA concentrations with age, sex, APOE ε4 status, and Aβ status. After false discovery rate (FDR) adjustment (q > 0.20), no significant associations were observed. Nominal trends (*p* < 0.05) indicated slightly higher butyric acid (*p* = 0.041) and valeric acid (*p* = 0.037) levels in males compared with females, suggesting minor sex-related differences in SCFA production (Figure 1B; Supplementary Table S2). Age and APOE ε4 status were not associated with any SCFA concentrations (Figure 1A,C; Supplementary Table S2). Overall, these results indicate that demographic and genetic factors exert minimal influence on fecal SCFA levels within this cognitively unimpaired cohort.

### 3.3. Stratified and Interaction Analyses (Amyloid-Stage and Demographic Subgroups)

#### 3.3.1. Aβ Status (Aβ Low vs. Aβ High)

Fecal SCFA concentrations differed modestly between Aβ Low vs. Aβ High groups (Figure 1D). Although no associations remained significant after FDR correction, acetic acid levels were nominally higher in Aβ High participants (*p* = 0.049), suggesting subtle compositional differences linked to amyloid burden.

#### 3.3.2. Sex (Male vs. Female)

Males exhibited nominally higher concentrations of butyric and valeric acids (*p* < 0.05) (Figure 1B), consistent with regression model results; however, no associations survived FDR adjustment.

#### 3.3.3. APOE Ε4 Status (Carrier vs. Non-Carrier)

No differences in SCFA concentrations were found between APOE ε4 carriers and non-carriers (all q > 0.05) (Figure 1C).

#### 3.3.4. Age Groups

Correlations between age and SCFAs were non-significant (all *p* > 0.05; Figure 1A).

### 3.4. Correlation Between Gut Microbial Species and SCFA Concentrations (Whole Dataset and Stratified by Amyloid Stage)

**Figure 2:**
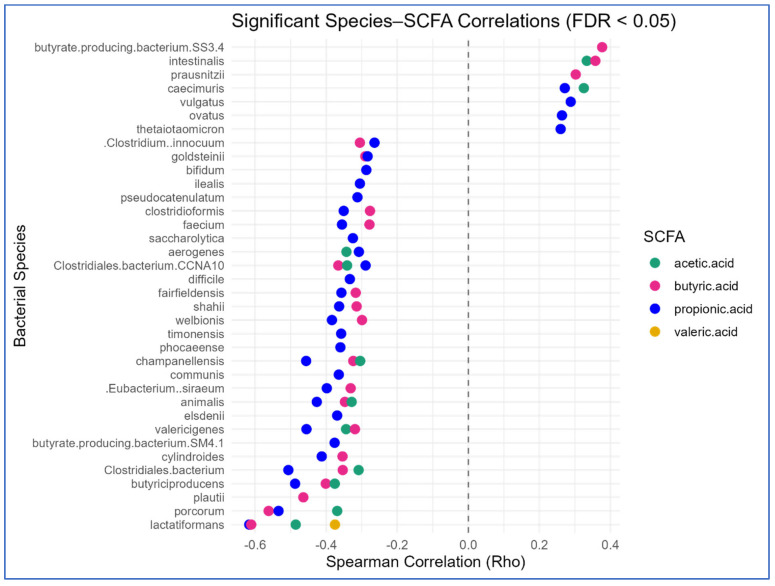
Gut microbial species significantly correlated with fecal short-chain fatty acids (SCFAs). Spearman correlation analysis was performed between the relative abundances of 103 microbial species and concentrations of six fecal SCFAs (acetic, propionic, isobutyric, butyric, isovaleric, and valeric acids). Only statistically significant species–SCFA correlations (FDR < 0.05) are shown. Dots represent individual species–SCFA pairs, with dot color indicating the SCFA type. Positive correlations are positioned to the right, and negative correlations to the left of the zero line (Rho = 0).

Spearman correlation analyses identified multiple FDR-significant associations between fecal SCFA concentrations and species-level microbial abundances (q < 0.05), spanning acetic, propionic, and butyric acids (Figure 2; Supplementary Table S2). Acetic acid showed the largest number of significant correlations, including positive associations with *Bacteroides ovatus* (ρ = 0.33, q = 0.024) and *Faecalibacterium prausnitzii* (ρ = 0.33, q = 0.023) and negative associations with *Bifidobacterium animalis*, *Lachnoclostridium scindens*, and *Enterocloster clostridioformis*. In addition, several taxa demonstrated significant correlations with propionic and butyric acid concentrations, although effect directions and magnitudes varied across species.

### 3.5. Species–SCFA Correlations Stratified by Aβ Status

**Figure 3:**
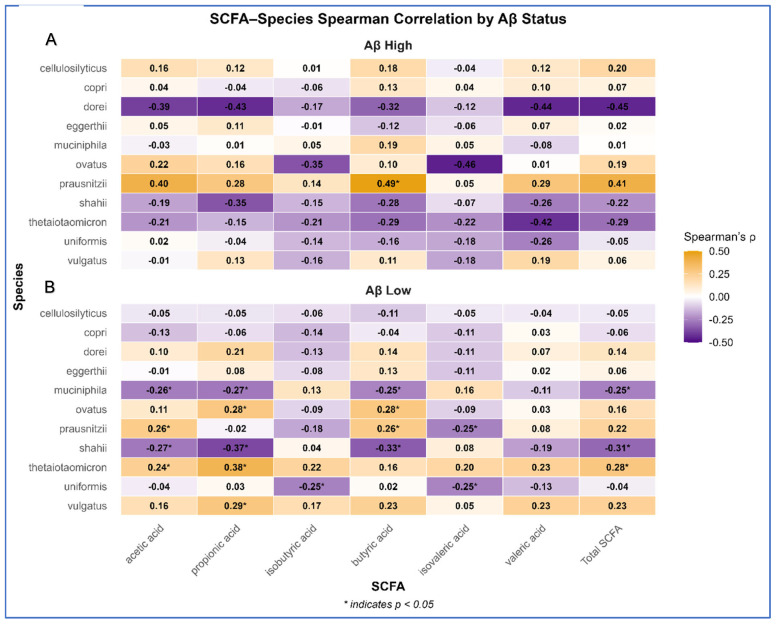
Spearman correlation heatmaps showing associations between gut microbial species and short-chain fatty acid (SCFA) concentrations, stratified by cerebral amyloid-β (Aβ) status. (**A**) Aβ High group exhibited stronger positive correlations for *B. thetaiotaomicron* and *C. cellulosilyticus*. (**B**) Aβ Low group showed broader associations across multiple SCFAs, notably *B. ovatus* and *B. shahii*. Positive correlations are displayed in orange, negative in purple; significant associations (*p* < 0.05) are marked with *.

Distinct correlation patterns were observed between microbial species and fecal SCFA concentrations across Aβ groups (Figure 3; Supplementary Table S3). In Aβ High participants, fewer but stronger positive associations emerged (e.g., *Bacteroides thetaiotaomicron* with butyric acid, ρ = 0.49; *Clostridium cellulosilyticus* with valeric acid, ρ = 0.45). In contrast, Aβ Low participants showed broader but weaker associations, including *B. ovatus* and *B. shahii* with multiple SCFAs (ρ = 0.22–0.30). These findings suggest that microbial–SCFA interactions may differ functionally with cerebral amyloid burden.

### 3.6. Multivariate Pattern Recognition and Clustering (Whole Dataset; Amyloid as Grouping Factor)

**Figure 4:**
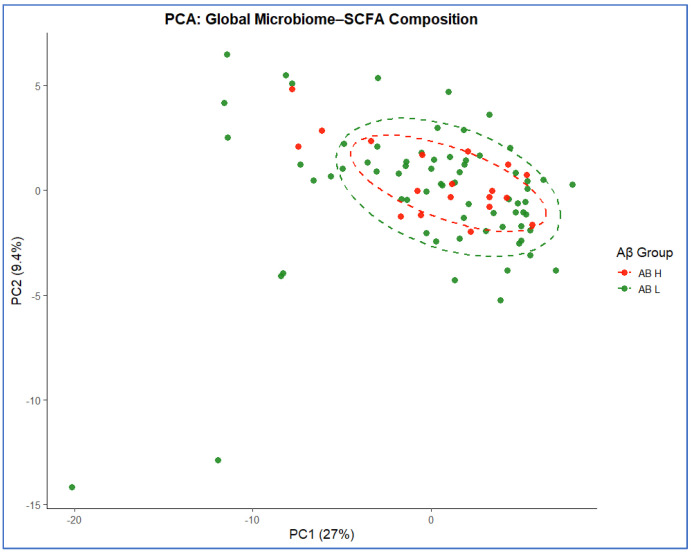
Principal component analysis (PCA) of global microbial–SCFA composition. PCA of CLR-transformed species and SCFA data illustrating partial overlap between Aβ High (orange) and Aβ Low (green) participants. Ellipses represent 68% confidence intervals. PC1 and PC2 explain 27% and 9.4% of the total variance, respectively.

**Figure 5:**
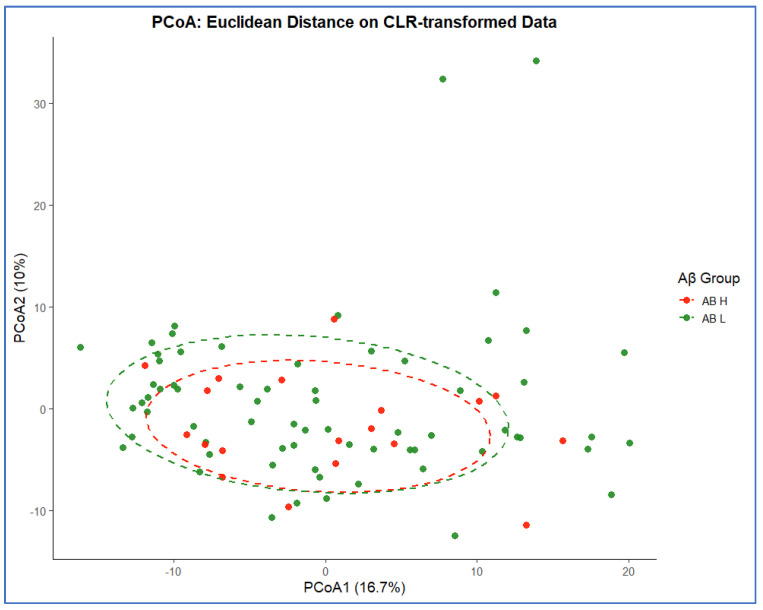
Principal coordinates analysis (PCoA) based on Euclidean distance. PCoA visualizes compositional dissimilarities in CLR-transformed data. Clusters of Aβ High (orange) and Aβ Low (green) groups overlap substantially, consistent with non-significant PERMANOVA (R^2^ = 0.056, *p* = 0.56).

Principal component analysis (PCA) and principal coordinates analysis (PCoA) were conducted on centered log-ratio (CLR) transformed microbial–SCFA data to assess global compositional structure. PCA revealed partial overlap between Aβ Low vs. Aβ High groups, with PC1 and PC2 explaining 27% and 9.4% of the total variance, respectively (Figure 4). Similarly, PCoA (Euclidean distance) showed overlapping clusters (PCoA1 = 16.7%, PCoA2 = 10%) with moderate within-group dispersion (Figure 5). PERMANOVA confirmed that Aβ status accounted for 5.6% of the total variance (R^2^ = 0.056, *p* = 0.56), indicating no significant overall compositional divergence when age, sex, and APOE were considered.

### 3.7. Canonical Correlation Analysis (CCA) Analyses (Whole Dataset; Amyloid Included as Covariate)

**Figure 6:**
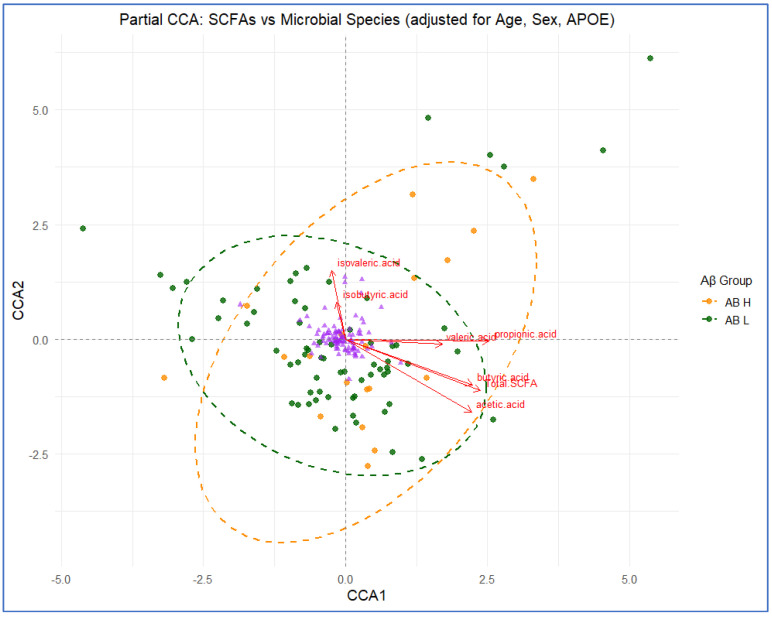
Partial canonical correspondence analysis (pCCA) of SCFA and microbial species composition adjusted for age, sex, and APOE status. Biplot shows constrained ordination of species (points) and SCFAs (red arrows). Aβ High (orange) and Aβ Low (green) ellipses indicate partial group separation (95% confidence). Total SCFAs and propionic acid contributed most strongly to Axis 1. Purple triangles are microbial species.

Partial CCA (pCCA) identified significant multivariate associations between SCFA profiles and microbial species composition after adjusting for age, sex, and APOE ε4 status (permutation *p* = 0.041). CCA1 captured the dominant constrained variance, revealing gradients corresponding to total SCFAs and propionic acid, which were significant contributors to species separation (*p* = 0.001 and *p* = 0.035, respectively) (Figure 6; Supplementary Table S4). Aβ High participants aligned more closely with butyric acid and total SCFA vectors, suggesting modest compositional shifts linked to amyloid status. Species such as *Faecalibacterium prausnitzii* and *Subdoligranulum* trended along butyric and valeric acid vectors, indicating potential metabolic coupling.

### 3.8. Mediation Analyses (Amyloid-Stratified Causal Framework)

**Figure 7:**
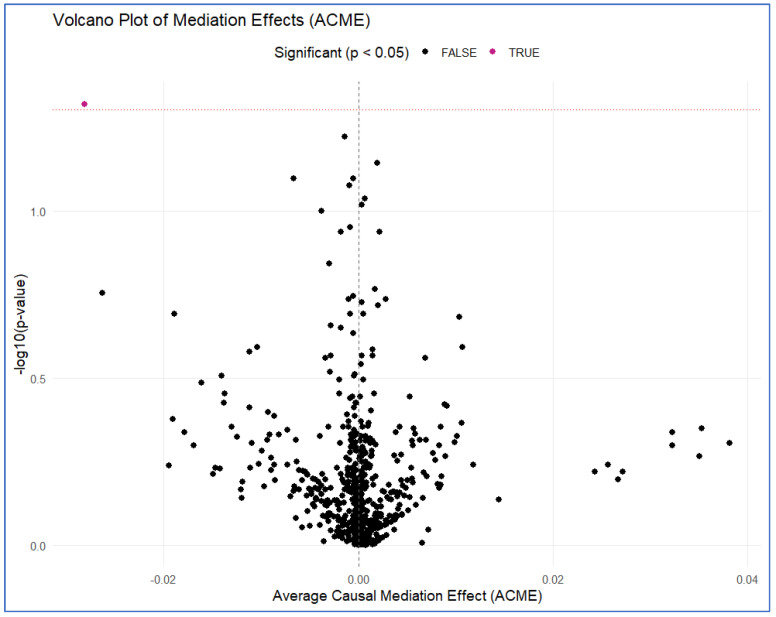
Volcano plot of the average causal mediation effect (ACME) across all SCFA–taxon pairs. Each point represents a taxon–SCFA model; the pink point denotes the only significant mediator (*p* < 0.05). Data cluster near zero ACME values, suggesting limited overall mediation.

**Figure 8:**
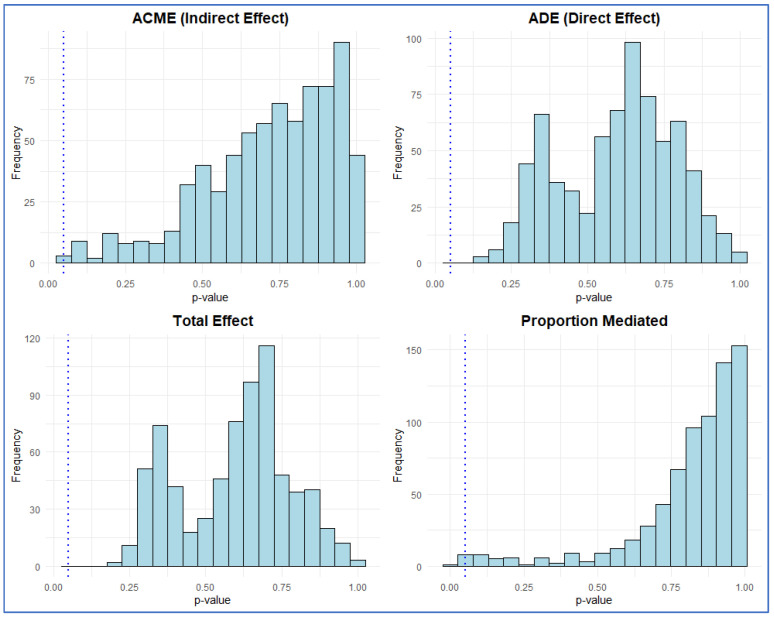
Distribution of *p*-values for mediation components. Histograms display *p*-values for ACME (indirect), ADE (direct), total, and proportion-mediated effects across all SCFA–taxon pairs. Blue dotted lines mark *p* = 0.05; most effects are non-significant.

Across 721 SCFA–taxon combinations, one species (*Clostridiales bacterium* within *Oscillospiraceae*) showed a nominally significant mediation of valeric acid’s relationship with Aβ status (ACME *p* = 0.048) (Figure 7; Supplementary Table S5). However, total and proportion-mediated effects were non-significant (Total *p* = 0.304; Prop *p* = 0.288), suggesting limited evidence for true mediation. Histograms of *p*-values for indirect (ACME), direct (ADE), total, and proportion-mediated effects showed right-skewed distributions, indicating that most models yielded non-significant results (Figure 8). The volcano plot demonstrated that nearly all taxa clustered around zero effect size, reinforcing the minimal mediating influence of individual microbes.

## 4. Discussion

This study examined the relationships between fecal SCFAs and gut microbial species in cognitively unimpaired participants stratified by cerebral amyloid-β (Aβ) status. Three key findings emerged. First, demographic and genetic variables (age, sex, and APOE ε4 status) did not appear to influence SCFA concentrations, suggesting that microbial–metabolite differences were not driven by these covariates. Second, acetic acid was the most abundant SCFA, and several commensal species, particularly *Faecalibacterium prausnitzii* and *Bacteroides ovatus*, showed strong positive associations with acetate. Third, Aβ High participants demonstrated altered microbial–SCFA interaction patterns, with fewer but stronger associations involving species such as *Bacteroides thetaiotaomicron* and *Clostridium cellulosilyticus*, alongside the enrichment of potentially pro-inflammatory taxa including *Lachnoclostridium scindens* and *Enterocloster clostridioformis*. Together, these findings indicate early functional shifts in microbial metabolism associated with amyloid burden, even before cognitive symptoms manifest. In addition to general inflammatory pathways, SCFAs may influence early AD-related processes through several mechanisms, including modulation of peripheral immune cell trafficking, effects on blood–brain barrier integrity, regulation of microglial activation states, epigenetic activity via histone deacetylase inhibition, and signaling through free fatty acid receptors (FFAR2/FFAR3) [22,23,24]. These pathways provide a plausible link between the microbial–SCFA patterns observed here and early amyloid-related biological changes. Additionally, the modest strength of most species–SCFA correlations reflects the fact that SCFA production arises from complex, multi-species fermentation processes, where substrate availability, cross-feeding, and host absorption collectively influence metabolite levels, and individual taxa therefore account for only a small fraction of the overall variance.

Interestingly, demographic and genetic variables, including age, sex, and APOE ε4 carrier status, did not show significant associations with fecal SCFA concentrations in this cohort. This absence of effect may reflect the relatively homogenous nature of the study population, which consisted of cognitively unimpaired, community-dwelling older adults with similar lifestyle patterns. SCFA levels are strongly influenced by diet, fiber intake, and medication use, which were not highly variable within this subset [25]. Moreover, the gut microbiome is known to stabilize in adulthood, with age-related changes becoming more pronounced only in later decades or in association with disease [26,27]. The lack of APOE-related differences also aligns with previous studies showing that this genotype primarily influences central lipid and amyloid metabolism, rather than peripheral microbial fermentation processes [28,29]. Collectively, these findings suggest that, within a metabolically stable and cognitively unimpaired population, host demographic factors exert only a modest influence on gut-derived SCFA profiles and that variation in these metabolites is more likely to reflect microbial composition and function than intrinsic host characteristics. It is also important to note that changes in microbial composition do not necessarily produce corresponding differences in fecal SCFA concentrations, as SCFA levels are influenced by dietary substrate availability, microbial cross-feeding, host absorption, and gut transit dynamics.

Recent studies have increasingly recognized the gut microbiota as a potential modulator of neuroinflammatory pathways in AD [5]. SCFAs, the principal fermentation products of dietary fiber, are central to the microbiota–gut–brain axis, exerting broad effects on metabolic and immune homeostasis [22,30,31]. In Aβ Low individuals, the broader range of positive correlations observed between multiple bacterial species and fecal SCFA concentrations, particularly acetate, propionate, and total SCFAs, suggests a functionally diverse and metabolically stable gut ecosystem. Taxa such as *Faecalibacterium prausnitzii* and *Bacteroides ovatus* are recognized for their established roles in producing anti-inflammatory metabolites and supporting mucosal health [32]. Their capacity to generate butyrate and other SCFAs, which enhance regulatory T-cell activity and strengthen epithelial barrier integrity, is consistent with previous reports linking these microbes to healthy aging, cognitive resilience, and protection against systemic inflammation [33,34]. Collectively, these findings reinforce the concept that a well-preserved fermentative network contributes to immune regulation and metabolic homeostasis in individuals without amyloid pathology [35,36].

In contrast, Aβ High participants demonstrated a narrower but more pronounced pattern of microbial–SCFA associations, primarily involving taxa with variable or pro-inflammatory metabolic profiles, such as *Bacteroides thetaiotaomicron* and *Prevotella copri* [37,38,39]. Although these species are capable of producing acetate and succinate, their enrichment in inflammatory conditions may indicate a shift from fermentation toward metabolic reprogramming under ecological stress [40,41]. The elevated acetate concentrations observed in Aβ High individuals are consistent with patterns reported in pro-inflammatory gut environments, where acetate may serve as an alternative energy substrate in response to reduced butyrate production. This adaptation may represent a compensatory mechanism within dysbiotic microbiota rather than a restoration of beneficial metabolic function [42,43].

However, the microbial profile of Aβ High participants revealed a contrasting pattern. Fewer taxa contributed to SCFA correlations, with stronger associations involving species such as *Bacteroides thetaiotaomicron*, *Prevotella copri*, and *Clostridium cellulosilyticus*. While these species are capable of carbohydrate fermentation, they have also been linked to mucosal inflammation and metabolic dysregulation in both gut and neurological disorders [39,44]. The predominance of acetate and valerate in Aβ High individuals may reflect metabolic reprogramming under dysbiotic conditions, where energy-yielding but pro-inflammatory fermentation pathways dominate [45,46]. This aligns with reports that increased acetate production can accompany gut inflammation and may contribute to glial activation when butyrate levels are reduced.

When examining overall community patterns, the data also showed that microbial profiles of Aβ Low and Aβ High groups were broadly similar, but subtle differences were apparent in how bacterial communities related to their metabolic activity. These differences were mainly influenced by the levels of butyrate and propionate (two SCFAs that are important for maintaining gut barrier function and regulating inflammation). This finding suggests that, even before major changes in the composition of the microbiome occur, functional shifts in metabolism may already be taking place [47,48].

Exploratory analyses examining possible causal links between microbial activity, SCFA production, and amyloid status suggested only a limited mediating effect. A single bacterial species from the *Oscillospiraceae* family appeared to partly connect valeric acid levels with amyloid burden, although this relationship was weak and not consistent across the dataset. Even so, this preliminary observation highlights the potential for specific microbial species to influence amyloid-related processes indirectly through their metabolic activity [49].

Valeric acid and its branched-chain counterpart, isovaleric acid, are mainly produced through the breakdown of amino acids during protein fermentation. An association involving these metabolites may therefore reflect shifts in microbial substrate preference; for example, a transition from fiber fermentation toward greater reliance on protein-derived pathways. Such a metabolic adjustment often occurs when beneficial fermenters decline or when the gut environment becomes more inflammatory [9,50].

Overall, these findings suggest that functional alterations in microbial metabolism may precede overt compositional changes. Rather than large-scale taxonomic shifts, subtle differences in how microbes generate or utilize metabolites like valerate could represent some of the earliest microbial signatures linked to amyloid-related metabolic imbalance.

The overall microbial patterns observed in this study are broadly consistent with previous research describing reductions in butyrate-producing taxa, such as *Faecalibacterium prausnitzii* and *Roseburia* spp., alongside increased representation of potentially inflammatory or opportunistic taxa including *Lachnoclostridium* and *Enterobacteriaceae* in individuals with AD and mild cognitive impairment [9,32]. Similarly, the diminished presence of *F. prausnitzii* and the enrichment of *Lachnoclostridium scindens* in Aβ High participants reflect a compositional imbalance previously linked to reduced gut barrier function, increased peripheral inflammation, and heightened amyloid pathology [51]. In this respect, the present findings extend earlier observations by showing that such microbial tendencies may be detectable even in cognitively unimpaired adults with elevated amyloid burden.

However, not all studies have reported reductions in SCFA production; some have found elevated fecal SCFA concentrations in early cognitive decline, possibly reflecting compensatory metabolic activity or differences in dietary fiber intake [49]. These inconsistencies may also arise from variations in sequencing depth, population diet, geography, and sample preservation methods, which can significantly affect microbial and metabolite profiles [52,53]. Nonetheless, when considered collectively, the literature supports a model in which the loss of butyrate-producing species and corresponding functional shifts in fermentation pathways precede neuroinflammatory activation and amyloid accumulation [28,54,55].

Importantly, this study contributes to the field by integrating species-level metagenomics and quantitative metabolomics in a preclinical cohort, enabling a more refined assessment of microbial–metabolite relationships prior to symptom onset. This approach highlights that not all SCFA–microbe interactions are beneficial; under dysbiotic conditions, some species may increase SCFA output through pathways that promote rather than suppress inflammation. These nuanced findings emphasize the importance of distinguishing between protective and maladaptive SCFA production when interpreting gut–brain interactions in the context of AD.

### Limitations and Future Directions

This study has several limitations. First, the cross-sectional design prevents causal inference; therefore, it remains unclear whether microbial or SCFA differences precede amyloid accumulation or reflect downstream effects of early Aβ pathology. Second, although shotgun metagenomics provides high-resolution taxonomic profiles, it does not directly assess microbial gene expression or metabolic activity. Future work incorporating metatranscriptomics and metabolomics would strengthen functional interpretation. Third, lifestyle and clinical factors known to influence the gut microbiome, including diet, medication use, and physical activity, were not fully controlled in this sub-study and may contribute residual confounding. Measures of gut-barrier function (e.g., zonulin, LBP) and circulating SCFAs were also not available, limiting conclusions regarding mucin degradation or systemic metabolic effects. Sex hormone measurements were not collected, and potential endocrine influences on SCFA concentrations could not be assessed within this dataset. Finally, participants were classified as cognitively unimpaired based on AIBL/WAMS criteria, but detailed neuropsychological test scores were not available; subtle cognitive changes cannot therefore be entirely excluded.

Despite these limitations, the findings provide early evidence of altered microbial–SCFA patterns in cognitively unimpaired individuals with elevated amyloid burden. Longitudinal and mechanistic studies, together with targeted dietary or microbiota-based interventions, will be essential to clarify the temporal sequence and biological relevance of these associations.

## 5. Conclusions

Taken together, the findings indicate that amyloid accumulation may be accompanied by an early reorganization of gut microbial metabolism, characterized by a selective loss of cooperative fermenters and the enrichment of stress-adapted or pro-inflammatory taxa. These functional alterations appear to disrupt the balance of SCFAs, particularly the ratio of butyrate to acetate, with potential downstream effects on immune regulation, intestinal barrier integrity, and neuroinflammatory signaling. Although the direction of causality cannot yet be established, the identification of key microbial and metabolic features associated with amyloid burden provides a foundation for the development of targeted interventions aimed at restoring microbial stability and metabolic resilience.

Future research should adopt longitudinal and mechanistic designs to determine whether these microbial metabolite changes precede or contribute to amyloid progression and cognitive decline. Integrating metatranscriptomic, metabolomic, and neuroimaging approaches will be crucial to clarify whether gut-derived metabolites exert direct effects on brain inflammation and amyloid dynamics. Ultimately, such studies may help define microbiome-based biomarkers and therapeutic strategies for early intervention in AD.

## Figures and Tables

**Table 1:** Participant characteristics and fecal SCFA concentrations by Aβ status.

Characteristic	CU Aβ Low (*n* = 68)	CU Aβ High (*n* = 19)	*p*-Value
Age, median (IQR)	76 (67–80)	81 (76–83)	**0.019**
Gender (Female), *n* (%)	48 (70.6%)	9 (47.4%)	0.107
Education, mean ± SD	14.43 ± 3.00	13.00 ± 2.47	0.057
APOE4 Positivity, *n* (%)	9 (13.2%)	7 (58.3%)	**0.026**
BMI, mean ± SD	25.41 ± 3.52	26.27 ± 4.37	0.467
Total SCFAs (µmol/g), median (IQR)	56.0 (42.8–68.3)	60.0 (36.9–77.0)	0.90
Acetic acid (µmol/g), median (IQR)	35.3 (26.3–43.3)	32.6 (24.5–47.5)	0.89
Propionic acid (µmol/g), median (IQR)	8.9 (6.1–11.7)	9.7 (5.9–13.5)	0.62
Isobutyric acid (µmol/g), median (IQR)	1.2 (0.84–1.60)	1.2 (0.94–1.45)	0.89
Butyric acid (µmol/g), median (IQR)	6.8 (2.5–9.2)	8.1 (3.0–13.0)	0.61
Isovaleric acid (µmol/g), median (IQR)	1.9 (1.2–2.5)	1.7 (1.45–2.35)	0.996
Valeric acid (µmol/g), median (IQR)	1.3 (0.82–1.63)	1.4 (1.05–2.05)	0.41

Data are presented as mean ± standard deviation for normally distributed variables and median (interquartile range) for non-normally distributed variables (e.g., SCFA concentrations). Group comparisons for continuous variables were performed using independent sample *t*-tests or Wilcoxon rank-sum tests as appropriate, and categorical variables were compared using chi-square tests. Abbreviations: Aβ, amyloid-beta; APOE, apolipoprotein E; CU, cognitively unimpaired; SCFAs, short-chain fatty acids.

## Data Availability

The data underlying this article were accessed from the Australian Imaging, Biomarkers, and Lifestyle (AIBL) study and the Western Australian Memory Study (WAMS) and are not publicly available due to ethical and privacy restrictions (including raw sequencing data and all derived metagenomic outputs). Access to AIBL data can be requested through the Expression of Interest (EOI) process outlined on the AIBL study website (https://aibl.csiro.au, accessed on 1 December 2025) in accordance with their data access procedures. Additional information regarding data access for AIBL and WAMS can be obtained by contacting the corresponding author.

## Supplementary Materials

The following supporting information can be downloaded at https://www.mdpi.com/article/10.3390/biom16010018/s1: Table S1: SCFA_Clinical_Correlations; Table S2: SCFA_Species_Correlations; Table S3: SCFA_Species_Correlation_by_AB_Groups; Table S4: CCA_SCFA_Biplot_Scores; Table S5: SCFA_species_spearman.
